# Unrelated toxin–antitoxin systems cooperate to induce persistence

**DOI:** 10.1098/rsif.2015.0130

**Published:** 2015-07-06

**Authors:** Rick A. Fasani, Michael A. Savageau

**Affiliations:** Department of Biomedical Engineering and Microbiology Graduate Group, University of California, Davis, One Shields Avenue, Davis, CA 95616, USA

**Keywords:** bistability, stochastic noise, heterogeneous population, multidrug tolerance, growth rate, environment

## Abstract

Persisters are drug-tolerant bacteria that account for the majority of bacterial infections. They are not mutants, rather, they are slow-growing cells in an otherwise normally growing population. It is known that the frequency of persisters in a population is correlated with the number of toxin–antitoxin systems in the organism. Our previous work provided a mechanistic link between the two by showing how multiple toxin–antitoxin systems, which are present in nearly all bacteria, can cooperate to induce bistable toxin concentrations that result in a heterogeneous population of slow- and fast-growing cells. As such, the slow-growing persisters are a bet-hedging subpopulation maintained under normal conditions. For technical reasons, the model assumed that the kinetic parameters of the various toxin–antitoxin systems in the cell are identical, but experimental data indicate that they differ, sometimes dramatically. Thus, a critical question remains: whether toxin–antitoxin systems from the diverse families, often found together in a cell, with significantly different kinetics, can cooperate in a similar manner. Here, we characterize the interaction of toxin–antitoxin systems from many families that are unrelated and kinetically diverse, and identify the essential determinant for their cooperation. The generic architecture of toxin–antitoxin systems provides the potential for bistability, and our results show that even when they do not exhibit bistability alone, unrelated systems can be coupled by the growth rate to create a strongly bistable, hysteretic switch between normal (fast-growing) and persistent (slow-growing) states. Different combinations of kinetic parameters can produce similar toxic switching thresholds, and the proximity of the thresholds is the primary determinant of bistability. Stochastic fluctuations can spontaneously switch all of the toxin–antitoxin systems in a cell at once. The spontaneous switch creates a heterogeneous population of growing and non-growing cells, typical of persisters, that exist under normal conditions, rather than only as an induced response. The frequency of persisters in the population can be tuned for a particular environmental niche by mixing and matching unrelated systems via mutation, horizontal gene transfer and selection.

## 1. Introduction

Persisters have traditionally been identified as subpopulations of cells that survive antibiotic treatment via epigenetic means. They were first recognized while treating *Staphylococcus* with penicillin [1] and were later identified as the source of multidrug tolerance in biofilms [2], making them responsible for 65% to 80% of bacterial infections [3,4]. Persisters have been implicated in the stubborn *Pseudomonas aeruginosa* infections to which most cystic fibrosis patients eventually succumb [5], as well as the oral *Candida albicans* infections common in cancer patients [6]. They may also explain the recurrence of *Mycobacterium tuberculosis* infections, responsible for 1.6 million deaths each year [7].

Persistence is not the result of a genetic mutation, but rather of a heterogeneous population. Modern single-cell studies have confirmed that persisters are rare, slowly growing cells [8], and that slowly growing cells are less susceptible to antibiotics [9]. More recent evidence suggests that slow growth is not necessary for nor a guarantee of persistence, but still increases the likelihood [10]. The mechanisms that provide the antibiotic tolerance are not fully understood, but one common path to persistence appears to be through the pervasive and varied toxin–antitoxin systems [11]. Toxin–antitoxin systems are genetic modules, commonly found in free-living bacteria, that generally consist of two co-produced and co-regulated components: a relatively stable toxin that inhibits cell growth and a more labile antitoxin that specifically neutralizes the toxin. Stress can upregulate the proteases that degrade the antitoxin, thereby freeing the toxin [12]. The overexpression of toxin can slow growth [13–19] and confer multidrug tolerance [18,20–22]. Conversely, multiple toxin–antitoxin systems are upregulated in persister-enriched samples [21,23]. In fact, the first gene tied to persistence was *hipA* [24], later identified as the toxic half of a toxin–antitoxin pair.

Toxin–antitoxin systems are found on the chromosomes and plasmids of most bacterial species and strains—the *Escherichia coli* K-12 genome boasts at least 36 [25] and the *M. tuberculosis* genome contains 88, more than any other human pathogen [26]. Yet, despite a growing understanding of the mechanisms underlying toxin–antitoxin systems, several important questions remain unanswered. What are their functions and how does each contribute to different cellular phenotypes or fates [27]? Why are there multiple types and apparently redundant systems in a single cell [28]? What is their coordinating signal [29]? Our previous work [30] answered some of these questions by forming a general model of the common type II toxin–antitoxin systems that target protein synthesis, and comparing the model behaviour to existing experimental results. Previous treatments addressed various aspects of toxin–antitoxin systems or persistent populations [8,31–36], whereas our analysis was the first to encompass them all, including molecular mechanisms of regulation, stochastic fluctuations, variable growth, and population dynamics, and to do so over a broad range of parameter values. We were able to describe a connection between the molecular mechanisms of toxin–antitoxin systems, the cooperation among systems to produce bistable expression, and the slow growth that is commonly associated with the persistent phenotype.

Our previous results [30] confirmed and explained genetic experiments [29] that revealed a characteristic and important relationship between the number of toxin–antitoxin genetic cassettes and the frequency of persisters that survive antibiotic treatment. Furthermore, these results suggested that although the specifics may vary, toxin–antitoxin systems are potentially bistable and can create a hysteretic switch between normal and persistent states. A bistable system can exhibit one of two stable behaviours under the same conditions, and it has become apparent that bistable genetic regulatory networks, when operating in noisy, fluctuating environments, can lead to heterogenous populations of cells. This can be seen in *Bacillus subtilis* genetic competence, spore formation, and swimming or chaining, as well as the persistent phenotype studied here [37]. We previously showed [29] how toxin–antitoxin systems that do not exhibit bistability alone can be coupled to produce the same effect. Moreover, the total number of toxin–antitoxin systems in a cell tunes the frequency of persisters, using the growth rate as the coordinating signal.

For tractability in treating the large numbers of toxin–antitoxin systems, our previous analysis considered the systems kinetically identical, even if their specific mechanisms differed. Indeed, the fact that the toxins inhibit protein production via diverse molecular methods and targets creates a multihit mechanism that is necessary for cooperativity, but not always sufficient—it can be readily shown that two systems with randomly chosen, differing kinetics do not always cooperate. Therefore, critical questions remain: whether or not toxin–antitoxin systems from different families—with similar motifs but dramatically different kinetics—cooperate, and if so, what the essential factors are that determine their cooperation.

Here, we remove the key restriction that provided tractability in the original model and account for multiple distinct toxin–antitoxin systems. We mix and match systems with kinetic parameters that vary over an order of magnitude, reflecting the parameter ranges that have been measured across several well-studied toxin–antitoxin families. The results extend our past findings, as well as offer new insight. In particular, multiple unrelated toxin–antitoxin systems cooperate via the growth rate, particularly in the presence of stochastic fluctuations, to robustly increase the size of the bistable region and the frequency of persisters. Furthermore, the size of the bistable region not only depends on the parameters of each system, but critically on their relative switching thresholds—the value of the stimulus at the inflection point of the toxin induction characteristic. As such, different toxin–antitoxin systems can be mixed and matched to provide the variation on which selection can act to tune the persister frequency for a given environmental niche.

## 2. Methods

### 2.1. Single-system model

**Figure 1.**
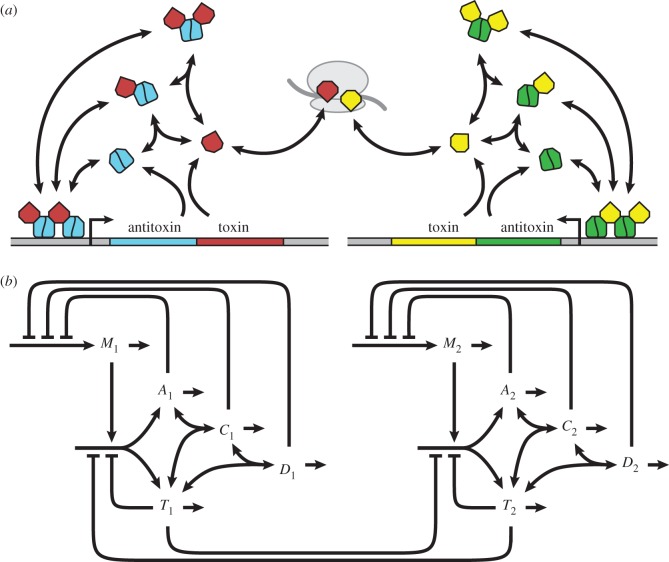
Model of coupled toxin–antitoxin systems. (*a*) The toxin and antitoxin are translationally coupled. The antitoxin binds and neutralizes the toxin, and can optionally bind a second toxin. The antitoxin dimer, either alone or in complex, autorepresses transcription by binding to one or more operators in the promoter region. The toxin enhances repression, in some cases via a bridging mechanism. Free antitoxin is relatively labile and degraded by various proteases (not shown). Free toxin usually inhibits some aspect of global translation. (*b*) *M*, mRNA; *A*, antitoxin; *T*, toxin; *C*, antitoxin bound to one toxin; *D*, antitoxin bound to two toxins. *A* and *T* are both translated from the polycistronic message *M*. Antitoxin alone or in complex—*A*, *C* or *D*—autorepresses transcription. Free toxin *T* inhibits translation, including its own. All species are degraded, and diluted by cellular growth. The degradation of *A* increases with proteolytic activity (not shown), and dilution is slowed when *T* inhibits global translation and growth (not shown). Either toxin—*T*_1_ or *T*_2_—can inhibit translation in both systems as well as the overall growth rate, which slows the dilution of all species.

Toxin–antitoxin systems use different protein structures and mechanisms, yet are consistent in overall architecture. Here, we describe a generic model for a large class of toxin–antitoxin systems: type II systems that target protein synthesis. Figure 1*a* depicts the common species and their interactions, and figure 1*b* represents a generic model for toxin–antitoxin systems, where *A* and *T* are the concentrations of free antitoxin and toxin, respectively. *T* can represent either monomeric or dimeric toxin, provided the toxin completely folds and dimerizes at physiological concentrations. We assume that the synthesis and degradation of the polycistronic message, *M*, as well as the formation and dissociation of the toxin–antitoxin complexes, *C* and *D*, are relatively fast compared with the rest of the system. As such, the dynamics of a single toxin–antitoxin system can be described by the following system of conventional differential equations:2.1
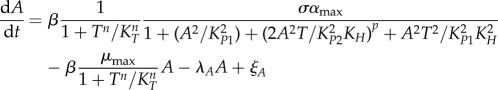
and2.2
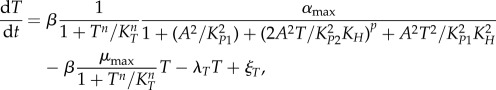
or by differential algebraic equations in the generalized mass action form [38]:2.3
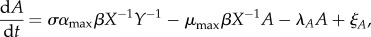
2.4
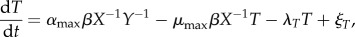
2.5
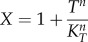
2.6
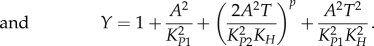
Equations (2.3)–(2.6) comprise a simplified model that retains the essential features of the version used in our previous work [30]. In both equations (2.3) and (2.4), the first and only positive term on the right-hand side represents protein synthesis. The multiplicative factor *σ* represents the translational coupling between toxin and antitoxin, and *α*_max_ is the maximum rate of protein synthesis. The second term represents protein loss due to dilution, where *μ*_max_ is the maximum growth rate constant. The third term in each equation represents protein loss due to active degradation, where *λ*_*A*_ is the degradation rate constant of antitoxin and *λ*_*T*_ is the degradation rate constant of toxin. Both protein synthesis and cellular growth are slowed by the toxic inhibition of translation *X*^−1^ [14,17], which is defined by equation (2.5). The impact of the free toxin *X*^−1^ follows a Hill equation with Hill number *n* and a concentration for half-maximal activity *K*_*T*_. Similarly, the independent, unitless parameter *β* reflects an external change that simultaneously lowers protein synthesis and cellular growth, or *α*_max_ and *μ*_max_. Protein synthesis is also proportional to the fraction of unbound promoter, *Y*^−1^, defined by equation (2.6). In most well-studied toxin–antitoxin systems, the operators are dissimilar, with one dominant, high-affinity site [39–44]. In this model, we ignore the weaker sites or, for the cooperatively binding complex, treat the sites in aggregate. The second term represents the dimerization of the antitoxin on the surface of the promoter, with a concentration of half-maximal binding at *K_P_*_1_. The third term represents the cooperative binding of the complex *C* to the promoter, with Hill number *p* and a dissociation constant *K_P_*_2_ that represents increased affinity. The complex *C* forms when a toxin binds the antitoxin dimer at one of two independent sites with a dissociation constant *K_H_*. The single toxin can bind to either site, and hence binds with twice the affinity, or 2/*K_H_*. The fourth term assumes that the complex *D* binds the operator with the same affinity as the bare dimer, or *K_P_*_1_. The complex *D* forms when two toxins bind the antitoxin dimer with overall affinity 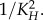

Studies over the past decade have confirmed the importance of stochastic fluctuations, or noise, in gene expression and genetic networks [45]. Noise is added to the model by augmenting equations (2.3) and (2.4) with additive noise terms, producing stochastic differential equations in Langevin form. *ξ*_*i*_ is a white noise term with zero mean, 〈*ξ*_*i*_(*t*)〉 = 0, and *δ*-autocorrelation, 〈*ξ*_*i*_(*t*)*ξ*_*i*_(*s*)〉 = *dδ*(*t* − *s*), where *d* is proportional the strength of the perturbation. Together, equations (2.3)–(2.6) form a tractable, generic model of toxin–antitoxin regulation.

### 2.2. Coupled-systems model

Experimental evidence indicates that one toxin–antitoxin system can trigger another, unrelated toxin–antitoxin system [46–48]. Our previous work suggested an indirect method of accomplishing such coupling [30]. If one system alone increases its own toxin concentration in response to a specific stress, and consequently slows protein production and growth, then another identical system may respond to the change in growth just as it would respond to a decrease in *β*. In this work, we extended the model to include a second, unrelated toxin–antitoxin system, as illustrated in figure 1. The extended model is described by equations (2.7)–(2.13), in which equations (2.7)–(2.9) describe the first system and equations (2.10)–(2.12) describe the second. Both subsets of equations mirror equations (2.3), (2.4) and (2.6) of the single-system model. The maximum growth rate constant *μ*_max_ and the parameter *β* are the same for both systems, whereas the other parameters can take on separate values. The systems are coupled by equation (2.13), where either toxin can affect both systems by attenuating global protein production and growth.2.7
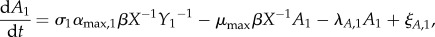
2.8
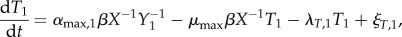
2.9
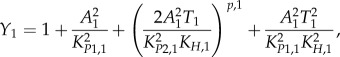
2.10
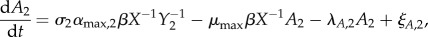
2.11
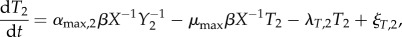
2.12
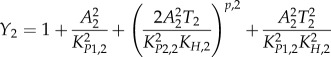
2.13
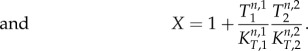


The known toxins target their own unique steps in translation [49] and it can be shown that their combined impact on translation is often multiplicative. For example, RatA blocks the association of the ribosomal subunits [50], whereas MazF cleaves mRNA [51]—decreasing both the concentration of functional ribosomes by half and the concentration of mRNA by half would reduce translation initiation fourfold. The coupled model nearly doubles the number of variables and parameters, but the same computational methods can be applied.

### 2.3. Parameter values

Apart from noise, the single-system model contains 11 parameters, which we estimated in previous work, based on the published data for six of the best-studied toxin–antitoxin systems: *kis-kid* (*pemIK*), *ccdAB*, *mazEF*, *phd-doc*, *relBE* and *yefM-yoeB* [30]. Table 1 includes the previously estimated parameter values. For technical reasons, in our previous work, we treated the kinetics of all toxin–antitoxin systems as identical. However, the published data indicate that toxin–antitoxin kinetics vary between families, sometimes over multiple orders of magnitude. Here, in addition to the original parameter estimates, we consider alternative sets of parameters, or alternative designs, that include various fold changes to the estimated values. The additional parameters are listed in table 1. The alternative sets of parameters vary over a broad range, but are not random. They were deliberately chosen for reasons we will make clear later in the text.

**Table 1.** Parameter estimates and alternative designs. Values listed under S1 were originally estimated in our previous work [30], based on the published literature for six well-studied toxin–antitoxin systems. Alternative designs S2–S7 include multiple twofold, fourfold or 10-fold changes—marked in bold—to the estimated parameters. The toxin and antitoxin half-lives, *t*_*T*_ and *t*_*A*_, as well as the maximum cellular doubling time, *t_*μ*_*, are shown here rather than their corresponding rate constants, which are trivially related by *λ*_*T*_ = ln 2/*t*_*T*_, *λ*_*A*_ = ln 2/*t*_*A*_ and *μ*_max_ = ln 2/*t_*μ*_*. In every design, *p* = 2, *n* = 2 and *t_*μ*_* = 30 min.

parameter	S1	S2	S3	S4	S5	S6	S7
*t*_*A*_ (min)	60	**120**	**240**	60	**120**	**240**	**120**
*t*_*T*_ (h)	48	**96**	**192**	48	**96**	**192**	**96**
*σ*	10	10	**2.5**	**2.5**	10	10	10
*α*_max_ (nM min^−1^)	1	1	1	**4**	1	**0.25**	1
*K_H_* (nM)	100	100	100	**400**	100	**25**	100
*K_P1_* (μM)	1	1	1	1	**10**	1	**0.1**
*K_P2_* (nM)	10	10	**2.5**	10	10	**2.5**	10
*K*_*T*_ (nM)	10	10	10	**40**	10	**2.5**	10

### 2.4. Computational procedures

We constructed and analysed the system design space using the Design Space Toolbox for Matlab v. 1.0 [52]. We simulated the deterministic model with the Matlab stiff solver, ode15 s. We simulated the stochastic model with our own implementation of the Euler–Maruyama method [53] in Matlab. All tests were performed using Matlab v. 7.8 (R2009a).

## 3. Results

### 3.1. Coupled cooperativity

**Figure 2.**
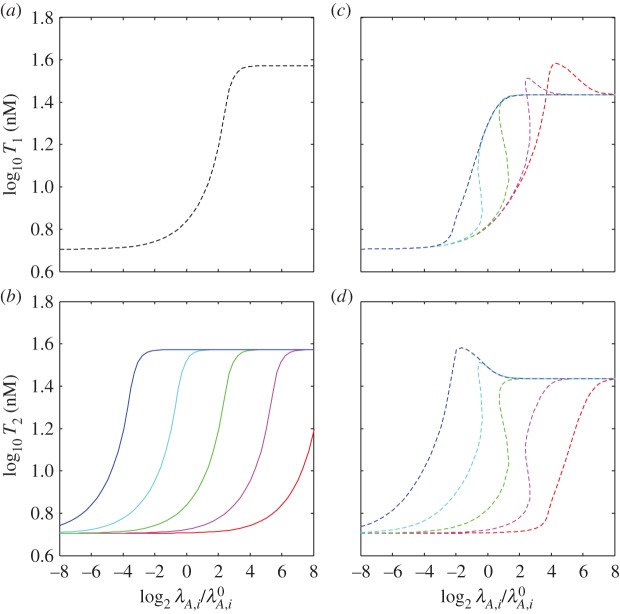
Toxin induction profiles for coupled and uncoupled systems. Steady-state toxin concentrations, *T*_1_ and *T*_2_, as a function of the changing rate constant for antitoxin degradation in both systems *λ*_*A*,*i*_. An increase in *λ*_*A*,*i*_ is commonly associated with an increase in stress. Changes in *λ*_*A*,*i*_ are measured as a fold change from the normal values 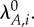 The proteolytic activity changes *λ*_*A*,*i*_ in both systems simultaneously, i.e. 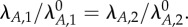 (*a*) Uncoupled reference system, using the originally estimated parameter values (dashed black). (*b*) Uncoupled additional systems using the same parameter values, except varying the normal rates of antitoxin degradation: 
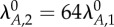
 (blue), 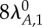 (light blue), 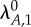 (green), 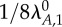 (pink) and 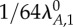 (red). The toxic thresholds, or the values of the stimulus at the inflection points of the toxin induction characteristics, are evenly spaced at approximately –3.7 (blue), –0.7 (light blue), 2.3 (green) and 5.3 (pink). (*c*,*d*) Steady-state toxin concentrations (dashed colour) when the reference system (*a*, dashed black) is coupled with each of the additional systems (*b*, solid colour). (*c*) The coupled reference system and (*d*) the coupled additional systems when 
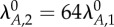
 (dashed blue), 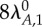 (dashed light blue), 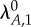 (dashed green), 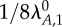 (dashed pink) and 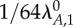 (dashed red).

The interaction of unrelated systems can be modelled by setting the parameters of equations (2.7)–(2.13) to represent two systems with different kinetics. Figure 2 shows the interaction of nearly identical systems where the difference in a single parameter value creates varying degrees of separation in their switching thresholds, or the value of the stimulus at the inflection point of the toxin induction characteristic. Figure 2*a* depicts the toxin profile of the first system, with the originally estimated parameter values, in response to changing *λ*_*A*_, which is commonly correlated with changing proteolysis and stress. Note that the system does not exhibit hysteretic bistability, whereas a nearly identical system in our previous work did. In our previous model, the toxin concentrations for half-maximal impact on growth (*K*_*T*1_) and protein production (*K*_*T*2_) were allowed to differ; here the toxin has the same impact (*K*_*T*_) on both, as defined by equation (2.5). This change eliminates the hysteretic region evident in our previous work and allows us to show here how systems that do not exhibit hysteresis individually can still cooperate via changes in growth rate to create hysteresis. Figure 2*b* shows the toxin profiles when a second, uncoupled system has the same, or nearly the same, parameter values, differing only in the normal rate constant for antitoxin degradation 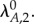 When the antitoxin is more stable (e.g. figure 2*b*, red), the proteolytic activity must increase further to reach the toxic threshold. Again, there is no bistability. Figure 2*c*,*d* illustrates the effect of coupling the first and second systems and increasing their rate constants for antitoxin degradation proportionally, as would be the case when both systems respond to the same protease. When the toxic thresholds are far apart, the system with the lowest threshold switches as it would in isolation (e.g. figure 2*d*, dotted blue) and the other system follows (e.g. figure 2*c*, dotted blue). The overall behaviour of the coupled systems is dominated by the most sensitive system. As their thresholds approach one another, the systems switch in unison, cooperation increases, and the switching becomes bistable and hysteretic (e.g. figure 2*c*,*d*, dotted green). The cooperation is robust: the antitoxin stabilities must differ more than eightfold to eliminate the hysteresis. However, these results describe the coupling of two systems differing by only a single kinetic parameter. Coupling, if any, between two completely unrelated toxin–antitoxin systems requires a more comprehensive analysis.

### 3.2. Design spaces

The model described by equations (2.3)–(2.6) is capable of exhibiting a rich phenotypic repertoire, and the individual phenotypes can be effectively enumerated and analysed within the system design space, as has been shown with other biological systems [54–56]. In design space, the behaviour of each phenotype is described by a dominant subsystem with a single, analytically defined steady state [52]. Note that equations (2.3) and (2.4) each have one positive term on the right-hand side, excluding noise, and multiple negative terms, while equations (2.5) and (2.6) each use multiple terms to define *X* and *Y.* Biologically, each term represents a process. For a given set of parameter values, one negative term or one defining term in each equation may be larger than the others, or dominate. If the smaller terms, or processes, are ignored, the behaviour of the remaining subsystem can be analysed using well-known techniques [57]. There are 32 possible cases, or combinations of dominant terms, and each case represents a potentially unique phenotype. In the figures that follow, each case will be depicted as a region in design space. Table 2 lists all of the regions that are visible in this work, along with a description of their dominant terms and some properties of their resulting phenotypes.

**Table 2.** Summary of characteristics for the regions in design space. As described in the text, each of the numbered regions represents a distinct case in which a different combination of terms creates a dominant subsystem that can be more effectively analysed. Here, the dominant terms, or processes, are described in each region. Antitoxin loss and toxin loss are found in equations (2.3) and (2.4), respectively. When the first negative term in either equation dominates, the principal form of loss is dilution, but when the second negative term dominates, the loss is primarily due to active degradation. Toxic inhibition hinges on equation (2.5), and when the first term dominates, the toxic inhibition of protein production is considered low; when the second term dominates, or the toxin concentration rises above *K*_*T*_, toxic inhibition is considered high. Transcriptional repression is described by equation (2.6), and when the first term dominates, the promoter is unbound, but when the third term dominates, the toxin–antitoxin complex acts as a strong autorepressor by cooperatively binding the promoter. Each region exhibits a distinct phenotypic behaviour, including the toxin concentration, growth rate and system stability, which are shown here. More detailed descriptions of the relevant regions and their significance can be found in the text.

region	dominant terms				system phenotype		
	antitoxin loss	toxin loss	toxic inhibition	repression	toxin concentration	growth rate	system stability
1	dilution	dilution	low	none	low	fast	stable
3	dilution	dilution	low	cooperative	low	fast	stable
7	dilution	dilution	high	cooperative	medium	moderate	stable
17	degradation	dilution	low	none	low	fast	stable
19	degradation	dilution	low	cooperative	low	fast	stable
21	degradation	dilution	high	none	high	slow	stable
23	degradation	dilution	high	cooperative	medium	moderate	unstable
27	degradation	degradation	low	cooperative	medium	moderate	stable
29	degradation	degradation	high	none	high	slow	stable
31	degradation	degradation	high	cooperative	high	slow	stable

**Figure 3.**
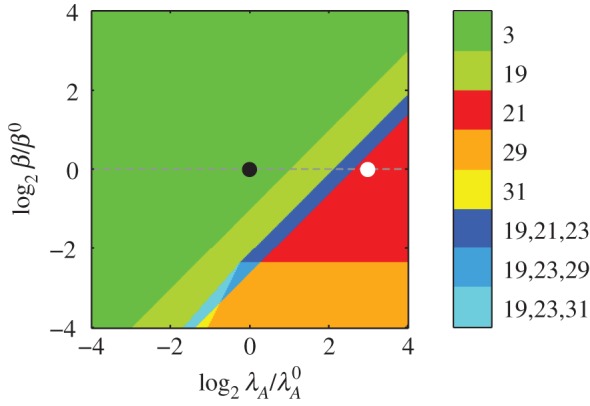
System design space for a changing rate constant of antitoxin degradation *λ*_*A*_. Each coloured region represents a different dominant subsystem, or phenotype. The axes represent a fold change in the parameters relative to the normal operating point (black circle) in Region 3. Holding the other parameters constant (dashed line) and increasing the rate constant of antitoxin degradation eightfold to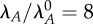 moves the system to a new operating point (white circle) in Region 21. See table 2 for additional information regarding the regions.

**Figure 4.**
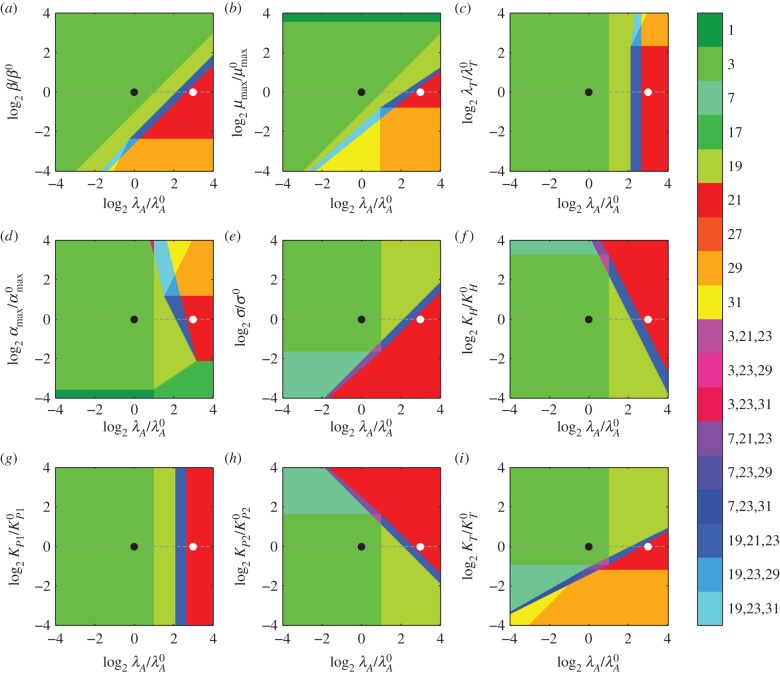
System design spaces for a changing rate constant of antitoxin degradation *λ*_*A*_. Each coloured region represents a different dominant subsystem, or phenotype. The axes represent a fold change in the parameters relative to the normal operating point (black circle) in Region 3. Holding the other parameters constant (dashed line) and increasing the rate constant of antitoxin degradation eightfold to 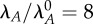 moves the system to a new operating point (white circle) in Region 21. (*a*–*i*) Each panel shows the variation of a different parameter on the vertical axis, along with the variation of *λ*_*A*_ on the horizontal axis.

Figure 3 depicts the design space over a wide range of parameter values. The normal operating point—defined by the originally estimated parameters—resides within Region 3, where dilution dominates in equations (2.3) and (2.4), free toxin is below its *K_m_* in equation (2.5), and transcription is mostly repressed by the cooperative complex, or the third term, in equation (2.6). The result is a phenotype with normal, or relatively fast, growth. Under stressful conditions, increased proteolytic activity increases the active degradation of the antitoxin, which is represented by moving to the right in figure 3. If the antitoxin half-life is reduced eightfold 
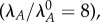
 the system moves from Region 3 to 21, where active degradation dominates the first equation, dilution still dominates the second equation, free toxin is above its *K_m_* in the third equation, and transcription is completely derepressed in the fourth equation. In other words, the toxin concentration rises and the growth rate falls. Notably, the system passes through a region of multiple phenotypes, or steady states. Our previous work showed that the intermediate regions represent a hysteretic transition between two stable steady states—high and low toxin levels—with an intermediate, unstable steady state [30]. Hysteresis, or bistability, is not evident in the toxin profile of figure 2*a*, but we also noted in our previous work that design space can overestimate the size of the hysteretic region, in which systems that do not exhibit hysteresis can still exhibit a supralinear, or ultrasensitive, profile. Indeed, figure 2*a* displays such ultrasensitivity. Furthermore, figure 3 indicates that the same behaviour should be expected over a wide range: if *β* is increased or decreased by twofold, an increase in *λ*_*A*_ would also move the system from Region 3 to Region 21, albeit at a different value of *λ*_*A*_. In fact, the boundaries are linear functions of the multiplicative parameter values [52], and therefore mathematically relate the changing threshold to the change in *β*. Simple observation indicates that halving *β* would halve the change required of *λ*_*A*_ to reach the threshold. In the same fashion, design space analysis can be used to explore the global behaviour of the nonlinear system over wide ranges of every parameter value—the impact of the other parameters is shown in the full set of design spaces depicted in figure 4. In nearly every case, a twofold increase or decrease in the parameter value does not change the system's hysteretic response to increasing *λ*_*A*_. As for the threshold of transition, increasing *α*_max_, *K_P_*_2_ or *K_H_* moves it closer to the normal operating point, whereas increasing *β*, *μ*_max_, *σ* or *K*_*T*_ moves the threshold farther away. Decreasing any of those parameters has the opposite effect. Changing *λ*_*T*_ or *K_P_*_1_ has little to no effect.

**Figure 5.**
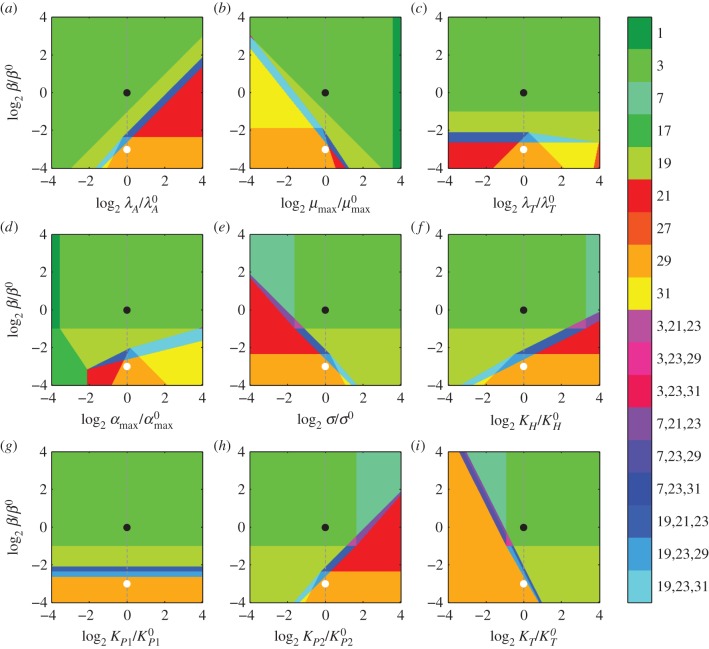
System design spaces for a changing *β*. Each coloured region represents a different dominant subsystem, or phenotype. The axes represent a fold change in the parameters relative to the normal operating point (black circle) in Region 3. Holding the other parameters constant (dashed line) and decreasing protein production and growth eightfold to *β*/*β*^0^ = 8 moves the system to a new operating point (white circle) in Region 29. (*a*–*i*) Each panel shows the variation of a different parameter on the horizontal axis, along with the variation of *β* on the vertical axis.

Decreasing *β*—a concomitant decrease in protein synthesis and growth—exhibits similar hysteretic behaviour, as shown in figure 5*a*. An eightfold decrease in *β* moves the system from Region 3 to Region 29, which is similar to Region 21 save that active degradation dominates in equation (2.4). In other words, the toxin concentration is high and the growth rate is even lower than in Region 21. The transition is hysteretic, similar to the transition induced by increasing proteolytic activity [30]. Furthermore, figure 4*b*,*d* shows that it is the decrease in maximum growth rate *μ*_max_, not the decrease in maximum protein production *α*_max_, that induces the change in phenotype. As in figure 4, the design spaces of figure 5 indicate how each parameter affects the threshold of hysteresis when lowering *β*. Increasing *λ*_*A*_, *α*_max_, *K_P_*_2_ or *K_H_* moves the threshold closer to the normal operating point; increasing *μ*_max_, *σ* or *K*_*T*_ moves the threshold farther away. Decreasing those parameters has the opposite effect. Changing *λ*_*T*_ or *K_P_*_1_ has little to no effect.

### 3.3. Steady states

In each case, the dominant subsystem can be analytically solved for a single steady state [52]. Of particular interest are the steady-state concentrations at the endpoints of the hysteretic switch, in Regions 3, 21 and 29. The concentrations, in terms of the independent parameters, are described by equations (3.1)–(3.6).3.1
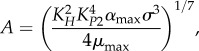
3.2
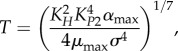
3.3
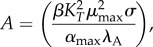
3.4
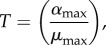
3.5
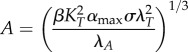
3.6
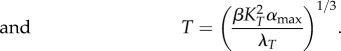


Equations (3.1) and (3.2) indicate that the steady-state concentrations in Region 3 are not dependent on *λ*_*A*_. However, as previously shown, an eightfold increase in *λ*_*A*_ shifts the system to Region 21, where equation (3.4) reveals that the toxin concentration is higher, but again independent of *λ*_*A*_. Together, equations (3.2) and (3.4) define the low and high toxin levels of a hysteretic switch that is sensitive to increased proteolytic activity. Similarly, an eightfold decrease in *β* shifts the system from Region 3 to Region 29, where the steady-state solutions are described by equations (3.5) and (3.6). Interestingly, the equations show that the concentrations remain dependent on *β*, so a further decrease in *β* will continue to decrease the toxin and antitoxin, albeit relatively slowly. Regardless, equations (3.2) and (3.6) define the low and high toxin concentrations of a hysteretic switch that is sensitive to the rate of protein production, or growth.

### 3.4. Alternative designs

The characteristics of the hysteretic switch depend on the parameter values, and the parameter values vary among toxin–antitoxin systems. The normal operating point is a representative estimate of measured parameter values in several well-studied systems [30] and is listed as design S1 in table 1. The table also describes pseudo-random alternative designs, or parameter sets, where each set includes multiple fold changes to the parameters of the normal operating point. Although none of the alternative designs represents a specific toxin–antitoxin system, the variations are typical of those seen in experimentally characterized systems [30]. For example, the antitoxin YefM has a measured half-life of 60 min [58], the value at the normal operating point, but Phd has a half-life of 120 min [59], a value used in alternative designs S2, S5 and S7. Furthermore, the promoter dissociation constant *K_P_*_1_ of Phd [39,60,61] is at least an order of magnitude lower than other well-studied systems [31,41,43,44,62–65], similar to the 10-fold decrease in alternative design S7. The translational coupling factor in *relBE* is 10 [66], the normal operating value, but for *kis-kid* the measured coupling factor is 2 [67], similar to alternative designs S3 and S4. The toxin and antitoxin typically bind tightly, but measurements of the dissociation constant *K_H_* vary: some estimates for *ccdAB*, *mazEF* and *relBE* are lower than the normal operating value of 100 nM [66,68,69], as is the value in S6, whereas the measured value for *yefM-yoeB* is 400 nM [58], the same value used in S4. Thus, the variation among the alternative designs is typical of the variation among toxin–antitoxin systems.

**Figure 6.**
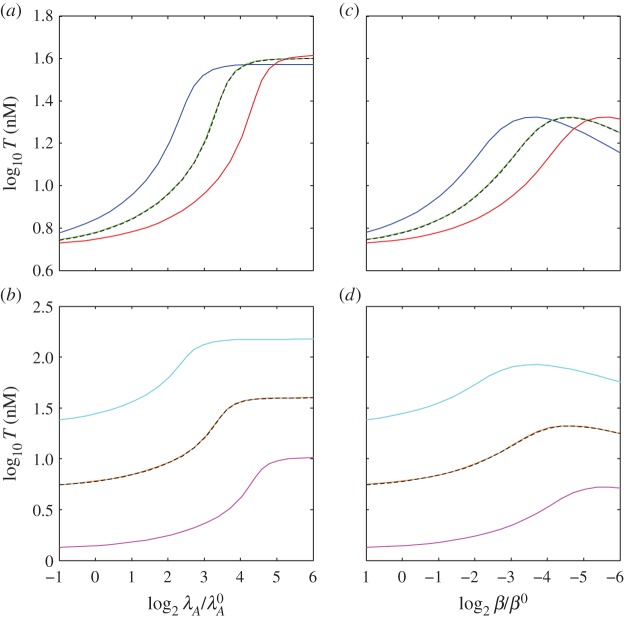
Toxin induction profiles for alternative designs. (*a*,*b*) Steady-state toxin concentration *T* as a function of the changing rate constant for antitoxin degradation *λ*_*A*_, in the alternative designs S1 (blue), S2 (green), S3 (red), S4 (light blue), S5 (orange), S6 (pink) and S7 (dashed black). Changes in *λ*_*A*_ are measured as a fold change from the normal value 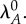 Note that the toxic profiles for S7 (dashed black), S2 (green) and S5 (orange) are identical. Also, the toxic thresholds, or the values of the stimulus at the inflection points of the toxin induction characteristics, are approximately the same for S1 (blue) and S4 (light blue), as well as for S3 (red) and S6 (pink), even though the toxin concentrations vary over different ranges. (*c*,*d*) Toxin concentration *T* as a function of *β* for the same alternative designs.

The panoply of design spaces in figures 4 and 5, together with equations (3.2), (3.4) and (3.6), describe the influence of each parameter on the relevant characteristics of the switch: the low and high toxin concentrations and the threshold of transition between them. The information can be used to predict the behaviour of the alternative designs. In fact, the alternative designs were chosen to exhibit specific variations in the toxic profiles, as shown in figure 6. Alternative designs S1, S2 and S3 were chosen to vary the threshold of toxic switching while maintaining approximately the same low and high toxin concentrations, which can be seen in figure 6*a*,*c*. Alternative designs S4, S5 and S6 were chosen to change both the toxic range and the toxic threshold, which can be seen in figure 6*b*,*d*. Designs S2, S5 and S7 were chosen to exhibit identical toxic profiles, despite their kinetic differences. Furthermore, figure 6 shows that none of the toxic profiles exhibits bistability, or more than one steady state along the curve, which is a result of the relatively low toxic impact *n*. However, the systems exhibit switch-like behaviour between two states and behave as designed, illustrating the potential power of design space to inform the forward engineering of biological systems.

### 3.5. Heterogeneous cooperativity

**Figure 7.**
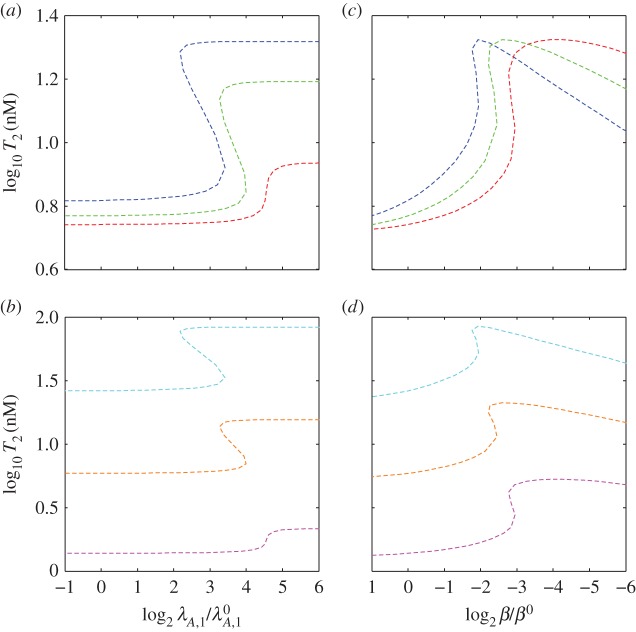
Toxin profiles for coupled alternative designs. (*a*,*b*) Steady-state toxin concentration of the second system *T*_2_ as a function of the changing rate constant for antitoxin degradation in the first system *λ*_*A*,1_. The first system is set to alternative design S7 and is matched with S1 (dashed blue), S2 (dashed green), S3 (dashed red), S4 (dashed light blue), S5 (dashed orange) and S6 (dashed pink). Changes in *λ*_*A*,1_ are measured as a fold change from the normal value 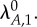 (*c*,*d*) Toxin concentration *T*_2_ as a function of *β* for the same couplings.

Figure 7 describes the effect of coupling the distinctly different alternative designs. The first system is set to the values for alternative design S7, and paired with each of the other alternative designs. *λ*_*A*,1_ is increased with no corresponding change in *λ*_*A*,2_, which models an increase in proteolytic activity that only affects the first system. The resulting steady-state toxin concentrations are shown in figure 7*a*,*b*. In every case, the stressed induction of the first system indirectly triggers the second system. Furthermore, in several cases, the systems together produce hysteretic behaviour, whereas none was observed for either system alone (figure 6). Figure 7*b* indicates that the cooperative effect depends on the relative toxic thresholds, but not the relative toxic ranges. The results are similar when *λ*_*A*,2_ is increased alone, or when *λ*_*A*,1_ and *λ*_*A*,2_ are increased together, as if both systems respond to the same protease (not shown). Figure 7*c*,*d* shows similar hysteretic behaviour when global growth and protein production are decreased via *β*. Decreasing *μ*_max_ alone also exhibits hysteresis (not shown). We showed in previous work that multiple identical toxin–antitoxin systems can cooperate to create hysteresis and increase the size of the bistable region [30]. Here, we show that extremely different toxin–antitoxin systems also cooperate when their toxic thresholds are similar, regardless of their toxic range. Similarly, the cooperation is robust to large changes in several parameter values, although the effect varies based on the relative toxic thresholds. As such, the mixing and matching of diverse systems via mutation, horizontal gene transfer and selection can generate a variety of switching profiles, of which some are optimal for a particular environmental niche.

### 3.6. Stochastic switching

**Figure 8.**
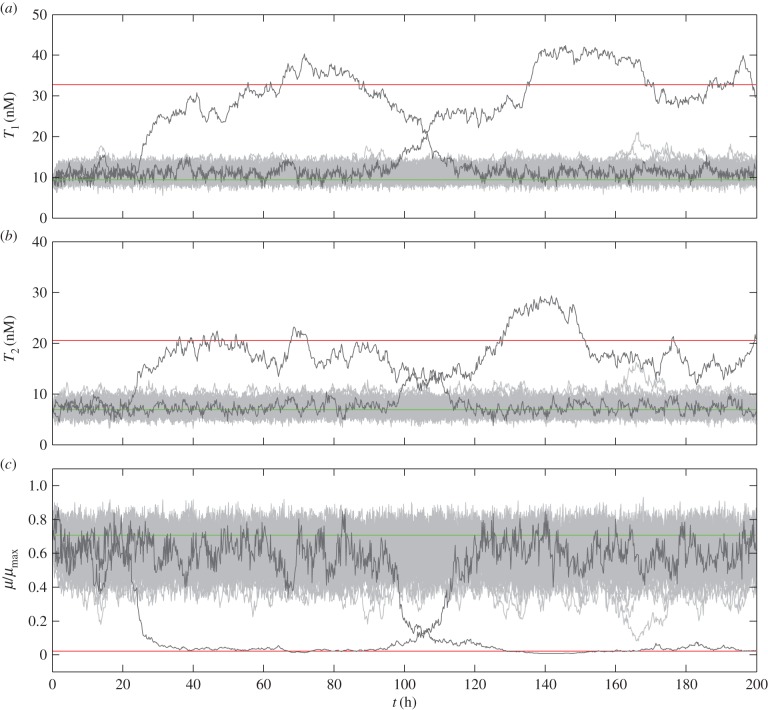
Stochastic simulation with spontaneous switching. Fifty thousand members of a population, with coupled alternative designs S1 and S7, were poised at the low-toxin steady state for 
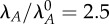
 and simulated with noise (*d* = 0.2). A random selection of 100 members (light grey) and two simulations of interest (dark grey) are compared with simulations without noise at the high steady-state toxin concentration (red) or the low toxin concentration (green). The concentrations of the first toxin (*a*) and second toxin (*b*) are different, but they switch at approximately the same time. (*c*) The growth rate is a function of both toxins, and spontaneously switches between normal (fast-growing) and persistent (slow-growing) states.

Any member of a population that operates within the bistable region should tend towards either the low or high toxin concentration, creating a distinctly bimodal population. Furthermore, our previous work showed that stochastic fluctuations can spontaneously switch cells from one state to the other [30]. However, those simulations assumed identical systems with correlated noise. In this work, we used the coupled model of equations (2.7)–(2.13), and stochastically simulated the unrelated systems with uncorrelated noise terms. We poised an entire population at the low toxin concentration, which corresponds to normal growth, and then added noise. Figure 8*a*,*b* shows that the toxin concentrations of the two systems fluctuated independently, generally near the low toxin concentration at which they were poised. The systems infrequently and spontaneously switched to fluctuate about the high steady state and appeared to switch together. As a result, figure 8*c* shows that individual members of the population infrequently and spontaneously switched to the slow growth, persistent state and, in at least one case, subsequently recovered. The transitions are infrequent, as is to be expected given that persister frequency in a population can be as low as 10^−6^ [24]. Numerous additional simulations exhibited the same behaviour, and mirror the stochastic behaviour we thoroughly tested and described in our previous work [30]. Here, the results confirm that the bistability introduced by unrelated toxin–antitoxin systems can also give rise to a dynamically changing subpopulation of persisters.

## 4. Discussion

Previous experimental work has shown that the frequency of persisters in a population is correlated with the number of toxin–antitoxin systems in the organism [29]. Our previous work provided a mechanistic link between the two. Using a simplified model where every toxin–antitoxin system was kinetically identical, we showed that the frequency of persisters is related to the width of the bistable region, which is in turn dependent on the number of toxin–antitoxin systems in the cell [30]. By extending the previous work, we are now able to show that the same result can be accomplished using a more realistic model that includes toxin–antitoxin systems with dramatically different kinetics. The bistable switch can be driven by proteolysis of the antitoxin, stochastic fluctuation or a change in the growth rate. The presence of bistability and the switching threshold itself are dependent on the other parameters of the system. Even if a single system lacks bistability, multiple systems can be coordinated by the growth rate to produce the same effect. Other work has shown a similar link between growth and bistability, where modulating the growth rate can create an implicit feedback loop, bistability and a heterogeneous population [70]. In our past work, the toxin–antitoxin systems were kinetically identical. The results here indicate that kinetically different systems—even systems that are dramatically different—can be coupled to produce bistability, and that the strength of the effect, or the size of the bistable region, is dependent on the relative switching thresholds of the individual systems.

Experimental work has shown that sufficient stress can induce persistence [71–73], which can also be seen in our model (figure 2). However, our model also shows how, under relatively normal conditions, stochastic fluctuations can spontaneously transition toxin–antitoxin systems to the persistent state. Even when the stochastic fluctuations in each system are uncorrelated, the toxin–antitoxin systems in a cell switch together, coupled by the growth rate. The spontaneous, coordinated switch to persistence describes how toxin–antitoxin systems can give rise to a bimodal population of normal and persistent cells. We predict that a persistent subpopulation would always be present, even under normal conditions, as a bet-hedging strategy to survive a catastrophic event. Indeed, there is some experimental evidence suggesting that this might be the case [8]. Interestingly, the switch back to the normal state is also spontaneous, and therefore the time spent in persistence is variable and unpredictable. It is possible that the emergence from persistence is just as important, if not more important, than entering it—persisters that emerge too early, before an environmental stress abates, will still perish. However, remaining dormant indefinitely is not a viable strategy either. A persistent subpopulation that survives catastrophe may employ a bet-hedging strategy of its own: individual members might randomly revert to normal and thrive if the stress is gone; if not, the remaining persistent population carries on.

Persister frequency in a wild-type population of *E. coli* is typically between 10^−6^ and 10^−5^ [24], but can vary by species, strain and environment—the frequency in a biofilm of *P. aeruginosa* may be as high as 10^−2^ [74]. Experimental results show that the frequency can be altered dramatically by varying the overall number of toxin–antitoxin systems in the cell [29]. Our model confirms those results and suggests that the effect is dependent on the average toxic impact of each system [30]. The average toxic impact of each system, in turn, is a function of their relative toxic thresholds. In other words, adding toxin–antitoxin systems with similar switching thresholds increases the persister frequency more than systems with relatively different thresholds. These results offer an explanation for the abundance, variation and apparent redundancy of unrelated toxin–antitoxin systems: the heterogeneous systems can be mixed and matched by mutation and horizontal gene transfer, creating populations with varying persister frequencies, each fit for a particular environmental niche.
